# “A Young Fellow by the Name of Guppy” (Dickens)

**Published:** 1910-01

**Authors:** 


"A YOUNG FELLOW BY THE NAME OF GUPPY" (Dickens).—Be sure to read Lydston's "Machine Methods of Defense" in this issue. The lightning strikes near home this time. He gives our little Yankee editor a merited rebuke. By the bye, the election for Secretary of the State Medical Association and editor of its journal takes place at Dallas at the annual meeting in May. We understand that the young man from the North will not be a candidate for re-election. During his incumbency as editor he has managed to create a split in the Association; has antagonized a large element of regular physicians who believe in the ethics of the Old

School; who believe in fair play and the square deal and who love truth and hate mendacity and hypocrisy, and who are disgusted with his sycophancy to the Octopus and will never affiliate with irregulars, negroes and quacks. He will make a virtue of necessity and get out. Like Carthage, he is doomed. Let him go back to Pennsylvania, to the shades of obscurity from which he sprang when he was imported to Fort Worth as a pious teacher in a Methodist theological school—a prayerful and lachrymose saint.

